# Supplementary material for: Functional role of brain-engrafted macrophages against brain injuries
Source: J Neuroinflammation. 2021 Oct 15;18:232. doi: 10.1186/s12974-021-02290-0 (PMC8520231; doi:10.1186/s12974-021-02290-0)
Supplement: Supplementary file 1 — Additional file 1. Supplementary Figures. Fig S1–S7. [file 12974_2021_2290_MOESM1_ESM.docx]

**Figure S1**


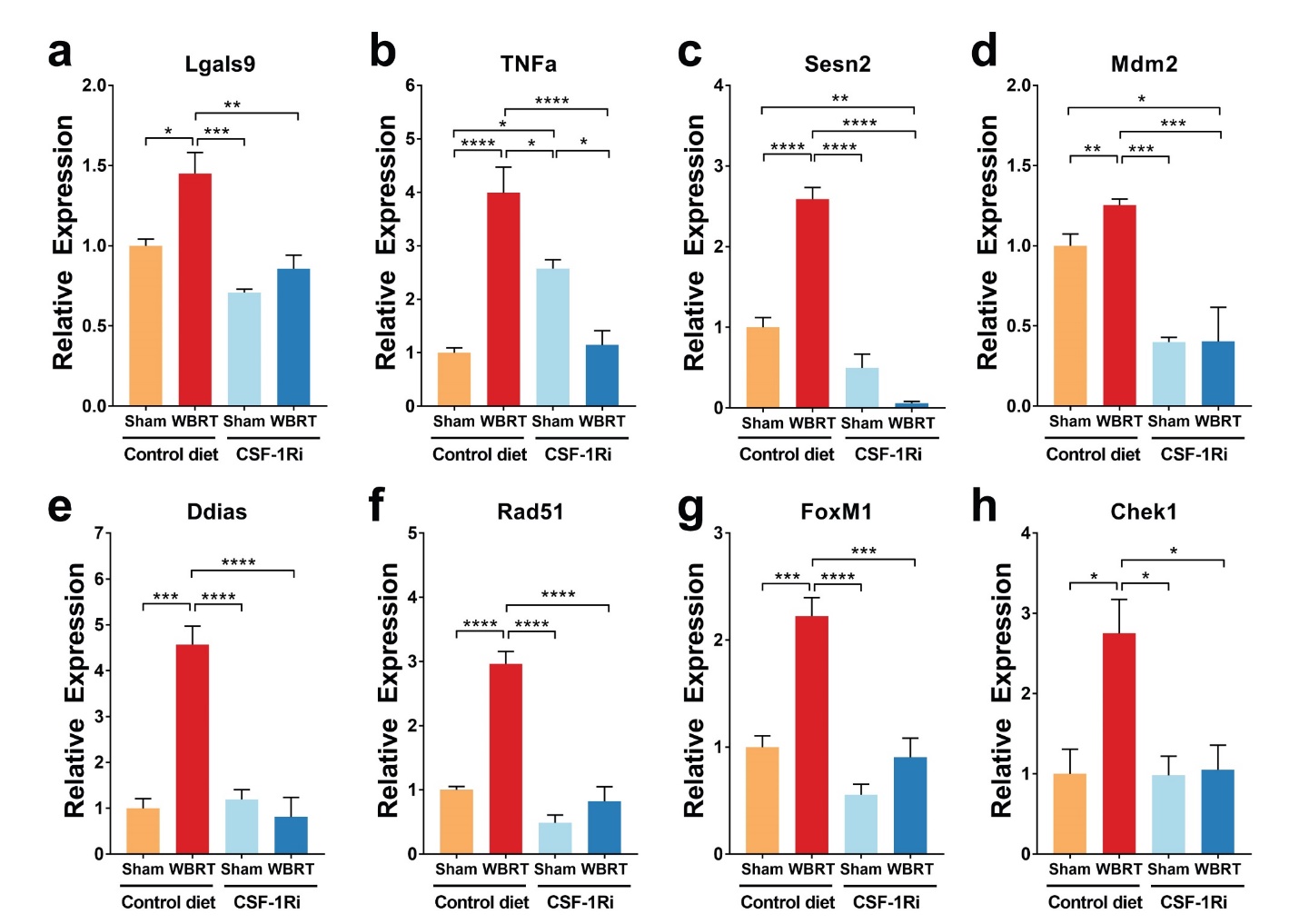


**Figure S1: qPCR validation of radiation-induced genes**

qPCR validation of radiation-induced genes. Genes from highly enriched GOBP terms were selected to validate RNAseq results. a and b, Toll-like receptor 3 signaling pathway: Lgals9 and TNFa. b and c, Regulation of response to reactive oxygen spieces: TNFa and Sesn2. d-e, Cellular response to ionizing radiation: Mdm2, Ddias and Rad 51. f-h, Regulation of double-strand break repair: Rad51, Foxm1 and Chek1. Statistical analyses were performed using two-way ANOVA with Tukey’s multiple comparisons test. *p<0.05, **p<0.01, ***p<0.001, ****p<0.0001. N = 4 – 6. The experiment was repeated twice with similar results.

**Figure S2**


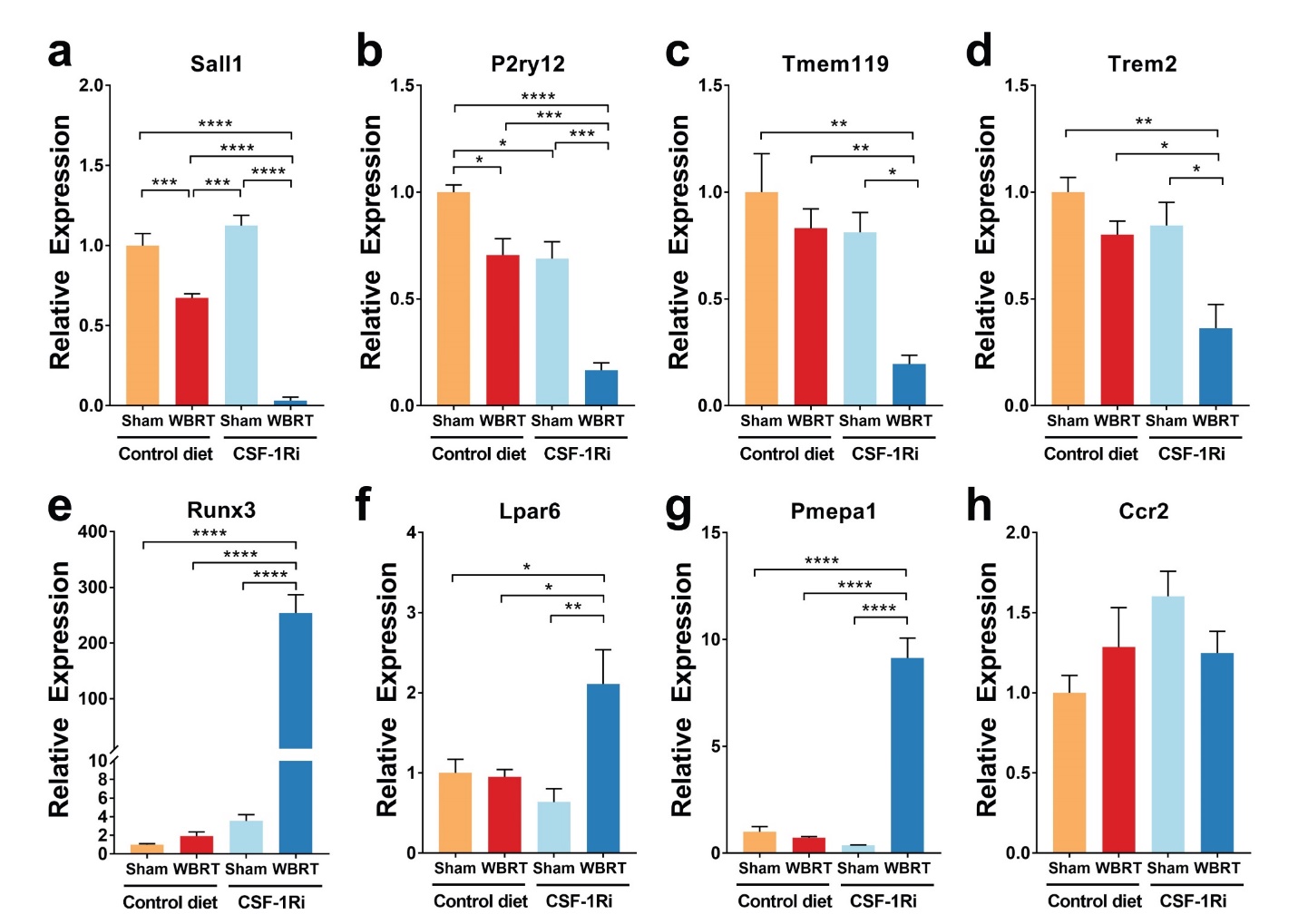


**Figure S2: qPCR validation of microglia- and monocyte-specific genes**. Selected genes that are known to highly express in microglia or monocytes were used to validate RNAseq results. **a – d** microglia signature genes *Sall1*, *P2ry12*, *Tmem119* and *Trem2* have lower expression levels in monocyte derived BEMs (CSF-1Ri + WBRT) compared to naïve microglia (control diet sham), irradiated microglia (control diet + WBRT) and repopulated microglia (CSF-1Ri sham). **e – f** monocyte signature genes *Runx3*, *Lpar6* and *Pmepa1* have higher expression levels in BEMs compared to other groups. Statistical analyses were performed using two-way ANOVA with Tukey’s multiple comparisons test. *p<0.05, **p<0.01, ***p<0.001, ****p<0.0001. N = 4 – 6. The qPCR experiments were performed in duplicates with similar results. Figures shown here are representative results from one experiment.

**Figure S3**


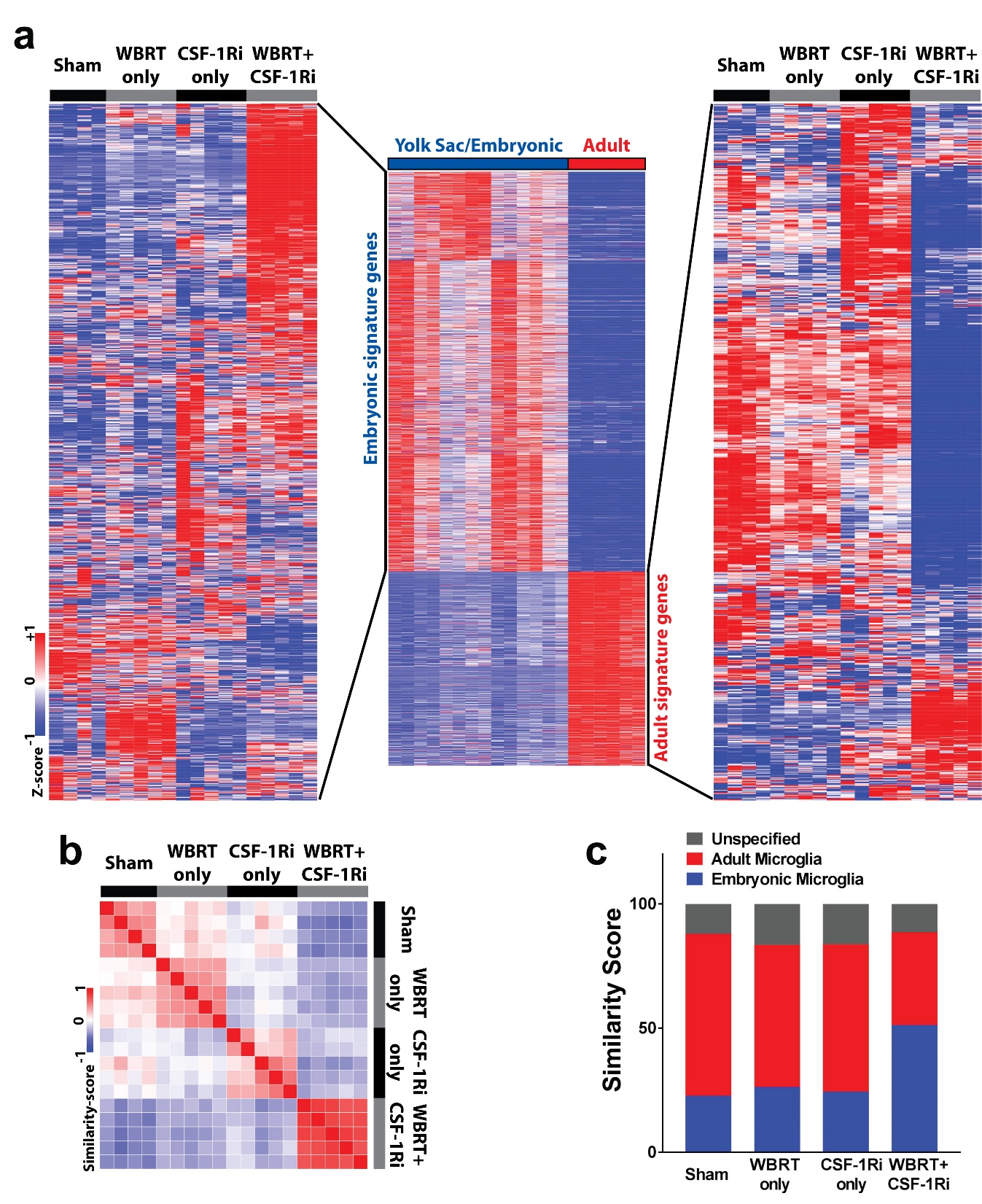


**Figure S3: Monocyte-derived BEMs after WBRT have embryonic microglia signatures**. **a** hierarchically clustered heatmaps to compare embryonic and adult microglia signatures across samples. Embryonic and adult signature genes were defined based on published dataset by Matchonitch and Winter et al. (Gene list and expression data in Additional file 4: Table S3). **b** Similarity matrix comparisons using defined embryonic and adult signature genes. **c** bar graph showing similarity scores to compare relative numbers of genes (shown as percentage of the defined list) that express in the same trends as embryonic or adult microglia in the Matchovitch and Winter dataset.

**Figure S4**


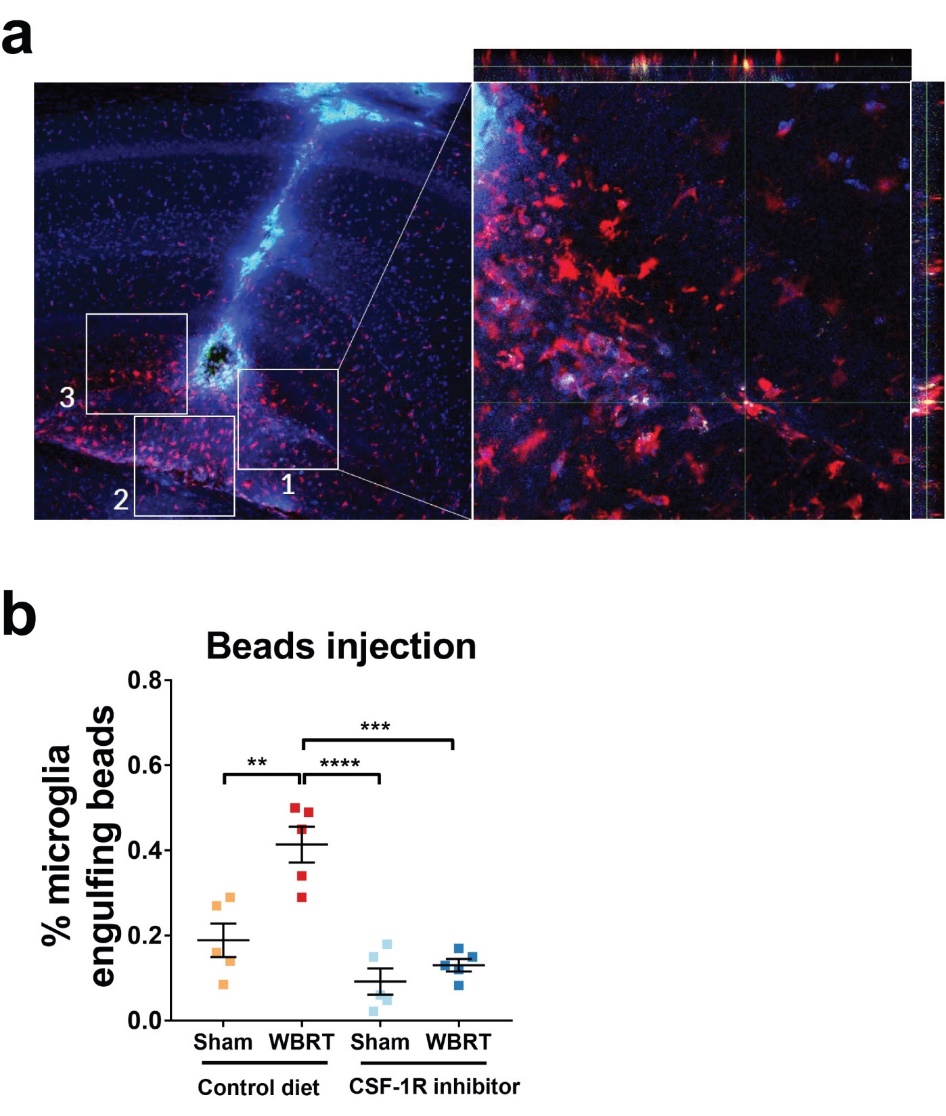


**Figure S4: Representative images of count window from phagocytosis assay by IF and result of in vivo beads phagocytosis assay by FACS**. A, representative images showing injection track of pre-stained synaptosomes and count windows. b, dot plot results of in vivo phagocytosis assay by FACS using fluorescent labeled beads. Statistical analyses were performed using two-way ANOVA with Tukey’s multiple comparisons test. **p<0.01, ***p<0.001, ****p<0.0001. N = 5.

**Figure S5**


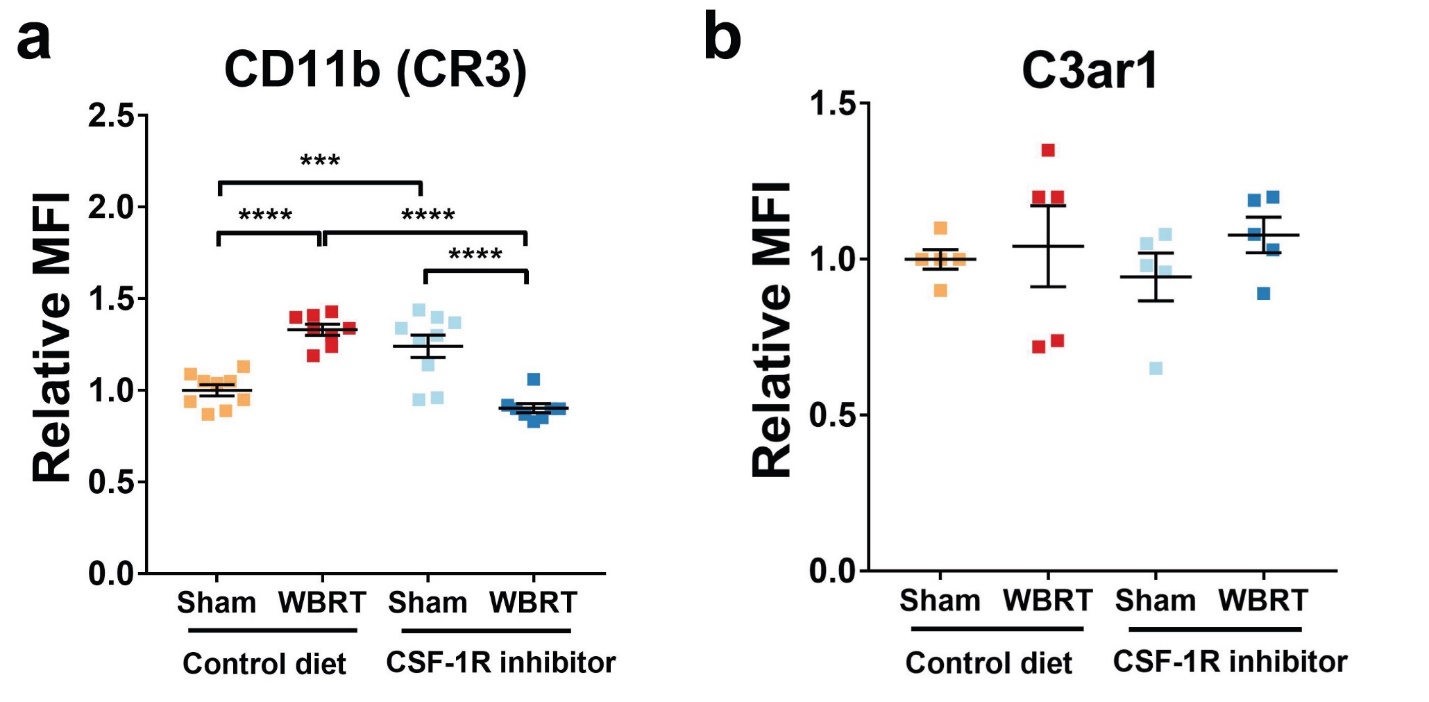


**Figure S5: Complement receptors CR3 and C3ar1 levels in microglia and BEMs.** a, dot plot of relative MFI of complement receptor CR3 (CD11b). b, dot plot of relative MFI of complement receptor C3ar1. Statistical analyses were performed using two-way ANOVA with Tukey’s multiple comparisons test. ***p<0.001, ****p<0.0001. N = 8 – 9 (a), N = 5 (b).

**Figure S6**


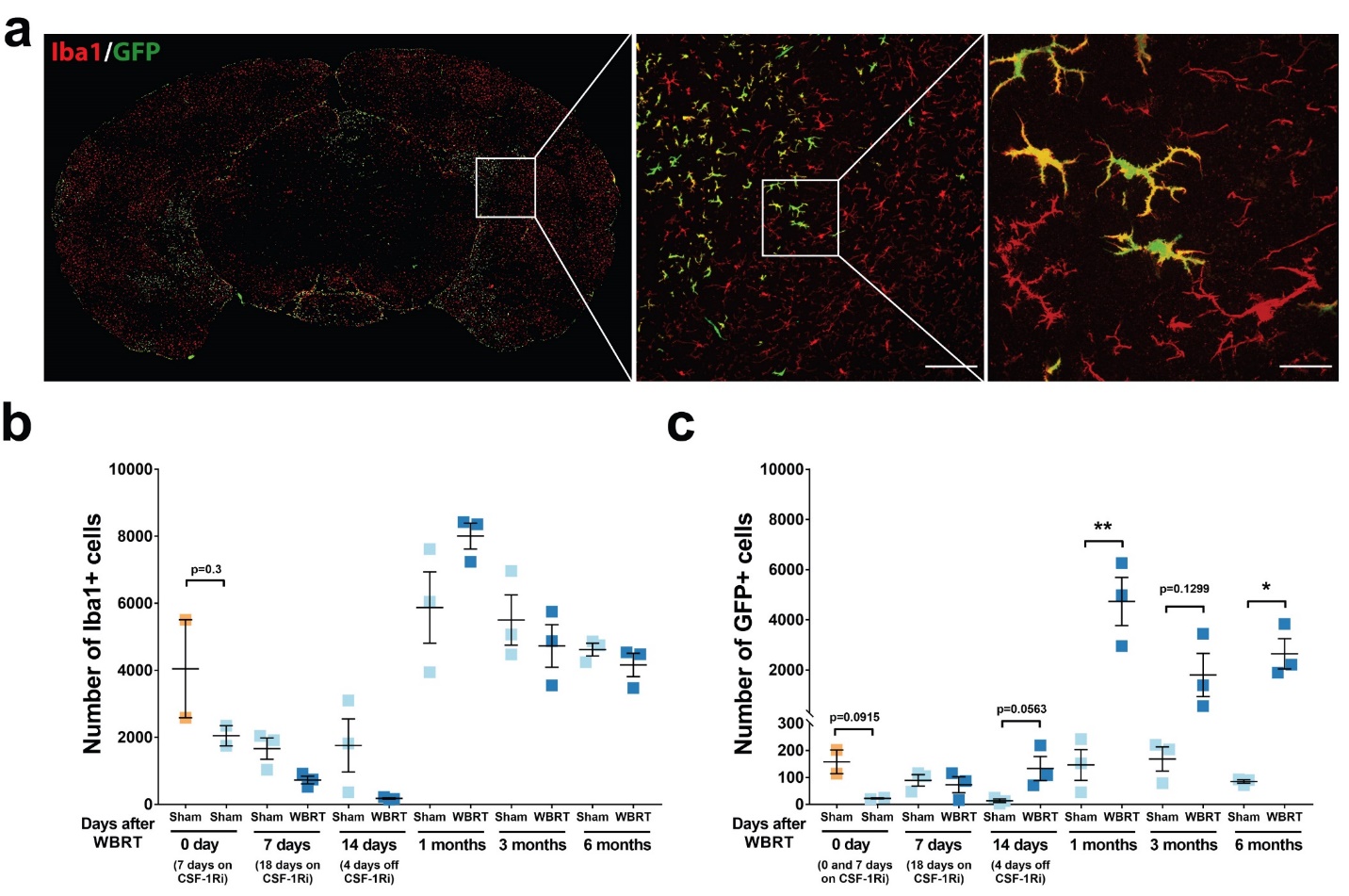


**Figure S6**: Quantification of microglia and BEMs in brains from bone marrow chimeras. **a** representative images of coronal section of whole brains from bone marrow chimeras. Scale bars = 100 μm (middle) and 20 μm (right). **b** dot plot of quantification results of Iba1 positive cells, each dot represents number of cells stained positive for Iba1 from a coronal whole brain section of an individual mouse. **c** dot plot of quantification results of GFP positive cells, each dot represents number of GFP positive cells from a coronal whole brain section of an individual mouse. Statistical analyses were performed using two-way ANOVA with Tukey’s multiple comparisons test. *p<0.05, **p<0.01. N = 2 (0 day sham on control diet) 3 (all other groups).

**Figure S7**


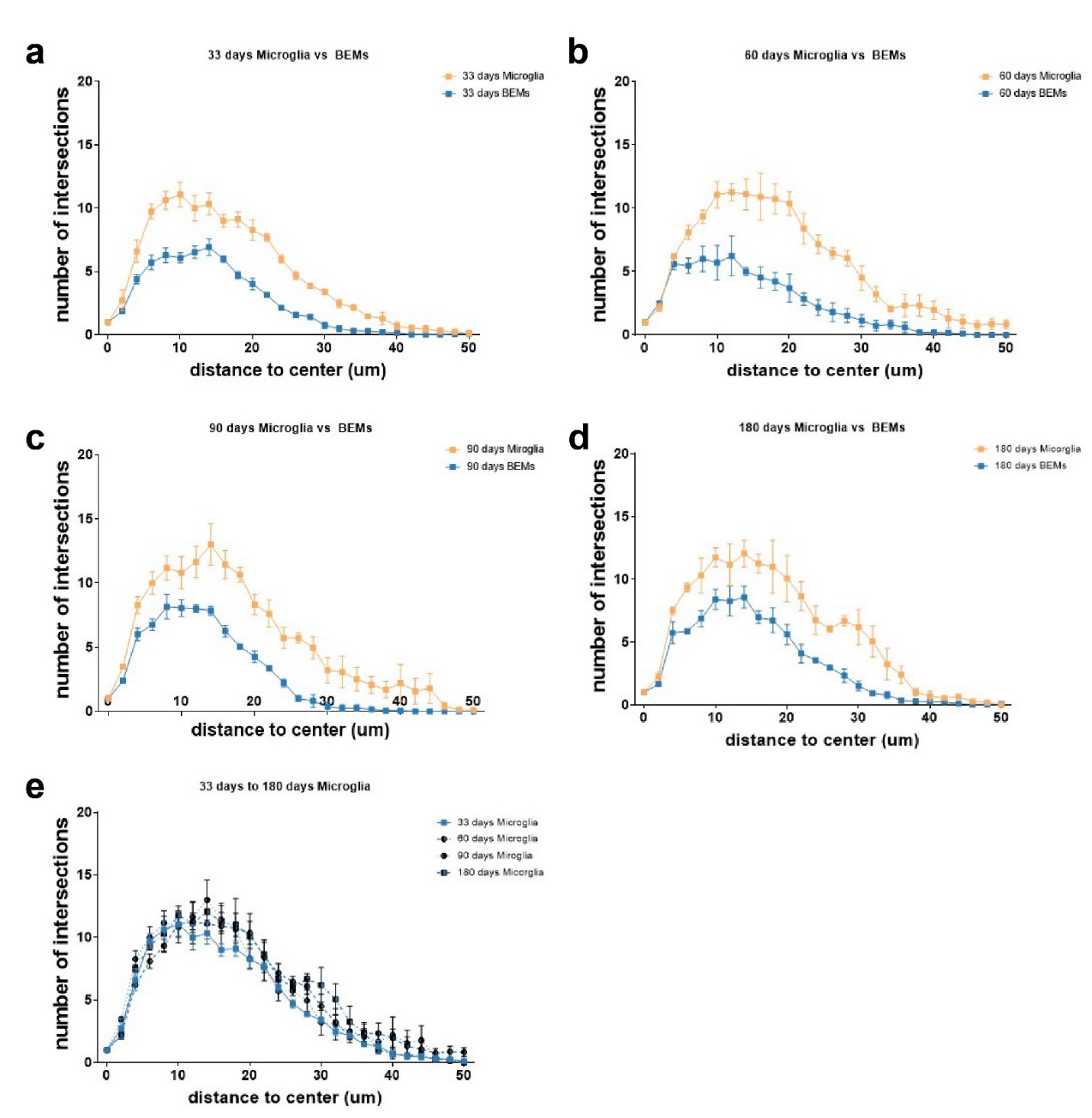


**Figure S7: Sholl analysis results of microglia vs BEMs over time**. a- d, comparison of Sholl analyses results between microglia and BEMs at 33, 60, 90 and 180 days after WBRT. e, Sholl analyses results of microglia at 33, 60, 90 and 180 days after WBRT. Statistics were performed using unpaired t-test at each distance point. n = 5 – 6.
